# Endoscopic Management of Strictures in Crohn’s Disease: An Unsolved Case

**DOI:** 10.3390/jcm13164842

**Published:** 2024-08-16

**Authors:** Gaetano Coppola, Chiara Principessa, Federica Di Vincenzo, Pierluigi Puca, Angelo Del Gaudio, Ivan Capobianco, Bianca Bartocci, Alfredo Papa, Giovanni Cammarota, Loris Riccardo Lopetuso, Franco Scaldaferri

**Affiliations:** 1 CEMAD—IBD Unit, Fondazione Policlinico Universitario Agostino Gemelli IRCCS, 00168 Rome, Italycapobianco_ivan@yahoo.it (I.C.);; 2 Dipartimento Universitario di Medicina e Chirurgia Traslazionale, Università Cattolica del Sacro Cuore, 00168 Rome, Italy; chiara99princ@gmail.com; 3 Dipartimento di Medicina e Scienze dell’Invecchiamento, Università degli Studi “G. D’Annunzio”, 66100 Chieti, Italy

**Keywords:** Crohn’s disease, Crohn, stricture, balloon dilation, EBD, stricturotomy, intestinal stent, fibrosis, nanoparticles

## Abstract

Crohn’s disease (CD) is a chronic inflammatory disease associated with a significant burden in terms of quality of life and health care costs. It is frequently associated with several complications, including the development of intestinal strictures. Stricturing CD requires a careful multidisciplinary approach involving medical therapy and surgery, still posing a continuous management challenge; in this context, endoscopic treatment represents a valuable, in-between opportunity as a minimally invasive strategy endorsed by extensive yet heterogeneous evidence and evolving research and techniques. This review summarizes current knowledge on the role of therapeutic endoscopy in stricturing CD, focusing on evidence gaps, recent updates, and novel techniques intended for optimizing efficacy, safety, and tailoring of this approach in the view of precision endoscopy.

## 1. Introduction

Crohn’s disease (CD) is a chronic inflammatory condition that can affect all gastrointestinal tract segments, with an intermittent and chronic course and increasing incidence [1]. Intestinal strictures represent one of the most frequent complications of CD, resulting from sustained transmural inflammation and abnormal extracellular matrix deposition and affecting approximately one-third of CD patients [2]. Two types of stricture can be identified: primary (de novo) and secondary (post-operative, anastomotic) strictures: primary strictures can develop in all segments affected by the disease, with the ileum being the most frequent site of onset, given the higher prevalence of disease-related inflammation in this segment and its smaller diameter compared to the colon [3,4].

Prompt multidisciplinary management of stricturing CD is required, considering the risk of bowel obstruction, the neoplastic potential [5], and the need to monitor upstream disease activity hidden proximally to the stricture. In this scenario, advanced therapy with biologics and small molecules may delay or reduce the need for repeated surgery in an bowel-sparing perspective [6]. Endoscopy stands out between medical therapy and surgery as a feasible, minimally invasive tool in selected cases, with an established role and still growing evidence in managing stricturing CD. Endoscopic balloon dilation (EBD) is the most extensively employed endoscopic procedure, being associated with a technical success rate exceeding 90% in most studies and a favorable safety profile for the treatment of short intestinal strictures [7]. Beyond EBD, in the last two decades, a wide range of alternative procedures have been gathering attention [8], with still limited data and a lack of standardization both in techniques and study designs [9]. In the era of precision endoscopy in inflammatory bowel disease (IBD), there is growing interest in advanced imaging techniques and artificial intelligence tools. These innovations aid in the detection of inflammation and dysplasia, as well as the identifications of molecular patterns for targeted interventions [10,11]. Both established and emerging therapeutic endoscopic tools hold promise for the management of persistent Crohn’s disease (CD) through increasingly microinvasive and personalized approaches. However, several challenges remain and there are many breakthrough opportunities to be explored.

**Figure 1:**
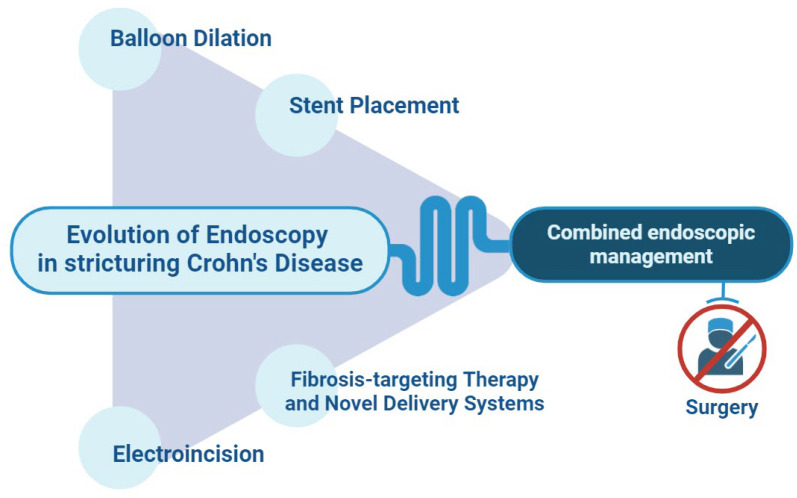
Evolution of endoscopy in stricturing Crohn’s Disease. The improvement of the known techniques alongside with the development of new technologies paves the way for an increasingly less invasive, combined endoscopic approach, with a view to tailored management, bowel-sparing strategy, and better quality of life.

Our review aims to build on established knowledge of endoscopic treatment of CD-associated strictures, identify evidence gaps, and highlight the latest advancements in the most intriguing techniques in development (Figure 1).

## 2. Methods

A bibliographic search was conducted using electronic databases including PubMed, Scopus, Embase and ClinicalTrials.gov. Search terms used included ‘Crohn’s disease’, ‘IBD’, ‘stricture’ combined with ‘EBD’, ‘balloon dilation’, ‘stricturotomy’, ‘strictureplasty’, ‘electroincision’, ‘intestinal stent’, ‘nanomedicine’, and ‘fibrosis’. Additional search terms for comparative studies included ‘comparison’, ‘outcome’, ‘efficacy’, ‘safety’, ‘long-term results’, ‘clinical trials’, ‘meta-analysis’ and ‘randomized controlled trials’. Bibliographies of relevant articles were searched manually; individual authors reviewed the titles and abstracts of the articles to assess their relevance to the study. Special attention was given to identifying comparative studies that directly evaluated the outcomes of EBD compared to other techniques in clinical trials and clinical practice for the treatment of CD-associated strictures. We ensured that the selected comparative studies provided robust data and clear results on regarding efficacy, safety, and long-term outcomes. This approach allowed us to gather relevant information to support the conclusions of our work.

## 3. Endoscopic Balloon Dilation

### 3.1. Endoscopic Balloon Dilation as the Standard Endoscopic Procedure for Short Strictures in Crohn’s Disease

EBD is the first described endoscopic treatment for intestinal strictures [12]. Since 1986, EBD has been applied for a wide range of gastrointestinal strictures and has been shown to be a straightforward, effective, and safe procedure, rapidly developing into the most used endoscopic treatment in stricturing CD upon proper selection of the patient, setting, and stricture type. Although extensive data are available, the heterogeneity of techniques and study designs made the adequate standardization and generalization of results challenging.

**Figure 2:**
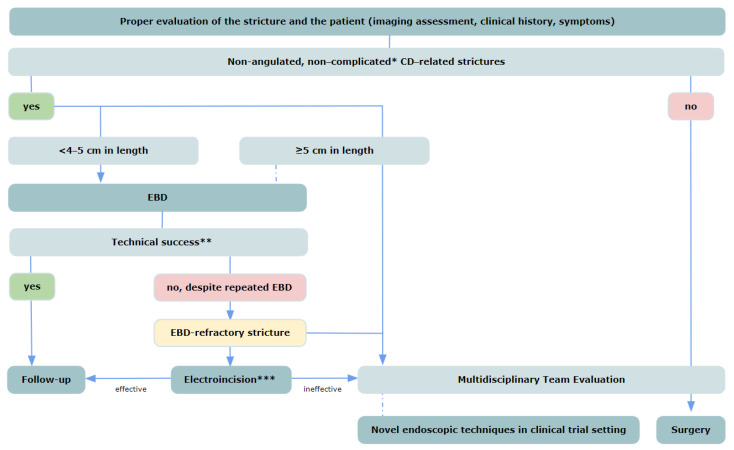
Endoscopic management of strictures in Crohn’s Disease based on the practice guidelines on endoscopic treatment for Crohn’s disease strictures [9]. CD, Crohn’s Disease; EBD, endoscopic balloon dilation. * No adjacent fistulae or abscesses; ** technical success in defined as post-procedural resistance-free passage of the endoscope through the stricture; *** only in centers with specific expertise.

The definition of the efficacy outcomes and the procedure technique widely vary both in study settings and clinical practice. According to the recent practical guidelines on endoscopic management of stricturing CD [9], a standardization of efficacy outcomes is advisable, in particular technical success (post-procedural resistance-free passage of the endoscope through the stricture, specifying the type of endoscope used), clinical efficacy (relief from occlusive symptoms at 6 months), and long-term efficacy (surgery-free survival at 1-year follow up). However, symptoms of CD-associated strictures may not reflect objective findings and the threshold for surgical intervention varies based on the patient and the surgeon’s preferences; moreover, the severity of the stricture and the persistence of symptoms might influence the need for an additional EBD [9]. The procedure technique is also not precisely defined and varies widely in the available studies. Currently, graded inflation is recommended over one-step inflation, as it allows proper inspection of the dilated tract after each controlled expansion and reduces the risk of bleeding and intestinal perforation [13]. Balloon sizes range from 12 to 20 mm and each dilation step varying from 20 s to 3 min in the available studies [12]. In a pooled analysis by Reutemann et al., no association between balloon size and surgery-free survival was found. Notably, patients undergoing dilations greater than 18 mm had an increased risk of surgery compared with 14 to 18 mm sizes, possibly due to the refractoriness of the disease in patients treated with larger balloons [14]. Whether all strictures can tolerate the same degree of dilation in a single episode, or instead, if features exist to stratify the dilation capacity of individual stenoses, remains to be clarified. In a systematic review including 33 studies from 1991 to 2013, with 1463 CD patients who underwent 3213 EBD procedures (62% anastomotic, 38% de novo strictures), a length < 5 cm was associated with a longer surgery-free interval after EBD; the rate of technical success was 89.1% and EBD resulted in clinical efficacy (remission of obstructive symptoms) in 80.9% of all patients, with no statistical difference between anastomotic and de novo strictures. However, at 2-year follow-up, 73.5% and 42.9% of patients underwent redilation and resective surgery respectively. A stricture length of <5 cm was associated with a surgery-free outcome with (HR 2.5; 95% CI 1.4–4.4; *p* = 0.002) and without (HR 2.4; 95% CI 1.3–4.2; *p* = 0.003) correction for stricture location, type of strictures, balloon caliber, steroid injection, and accessory endoscopic therapy. A cut-off for stricture length of <5 cm also showed a strong tendency to be associated with a redilation-free outcome (*p* = 0.06); however, no specific cut-off value for balloon size could be definitively recommended, although a larger balloon diameter was identified as a predictive factor for greater technical success [15]. Major complications, like perforation, bleeding, or surgery after dilation, occurred in 2.8% per procedure and 6.2% per patient. It remains unclear whether the efficacy and the complication rate may differ based on a different dilation strategy and technique (single-session versus multiple-session dilation, one-step versus graded dilation). The optimal dilation method for different types of strictures remains unclear. The number of dilations, the interval between dilations, and the length of follow-up highly varied widely between and, in some cases, within the included studies. Moreover, some patients may have undergone dilation in the absence of overt obstruction, which may limit the reliability and stratification of the results. Overall, EBD is indicated in CD patients with symptoms of bowel obstruction and non-complicated, non-angulated strictures shorter than 5 cm (Figure 2) [9]; graded dilation with balloons up to a maximum size of 18–20 mm is recommended [9]. Currently, there is no full agreement on whether or not to perform endoscopic dilation in asymptomatic patients; the patient’s symptoms do not necessarily correlate with the functional impairment caused by the stenosis, whereas the endoscopic treatment could delay or prevent symptoms and complication risk. Notably, symptomatic patients who undergo EBD typically have a poorer response to treatment and are at a higher risk of subsequent surgery compared to asymptomatic patients [9]; Similarly, pre-stenotic dilatation is associated with poor response to EBD and increased risk of bowel obstruction and surgery [16] despite representing the setting of the highest potential benefit of the endoscopic strategy when effective [17].

Beyond the many details to be clarified for proper standardization, recent comparative studies (Table 1) and ongoing trials promise to give new insights into the therapeutic role of EBD in CD. For instance, a prospective multicenter observational study is underway to evaluate the role of EBD in ameliorating mucosal and transmural inflammation of the prestenotic intestinal tract. Improved fecal flow and clearance of inflammatory mediators and microbiota could enhance the local gut microenvironment and reduce upstream inflammation (NCT04803916).

### 3.2. Intralesional Corticosteroid Injection

The role of intralesional corticosteroid injection following EBD remains unclear, aso two outdated prospective, randomized clinical trials have yielded conflicting results. East et al. compared local quadrantic injection of 40 mg of triamcinolone after EBD of short ileocolic anastomotic CD strictures (<5 cm) to saline placebo [23]; 1 in 6 patients in the placebo group required redilation compared to 5 in 7 in the steroid group, with a statistical trend towards a difference in time to repeat dilation, which was worse in the steroid group (*p* 0.06, HR 6.1). In a similar setting, Di Nardo et al. enrolled 29 pediatric CD patients with both de novo (17) and anastomotic (12) short strictures to receive or not intralesional quadrantic injection of 40 mg triamcinolone after EBD. In the placebo group, 5 and 4 of 14 patients required redilation and surgery at 12-month follow-up, respectively; in the experimental group only 1 out of 15 patients required redilation, and none required surgery. The groups differed significantly in time without re-dilation (*p* = 0.04) and surgery (*p* = 0.02) [24]. The results of these two trials are quite contrasting despite using similar injection techniques. However, a proper comparison between the two studies is limited by small sample sizes, different populations, and different stricture locations. In 2022, Feleshtynsky et al. displayed a new perspective on this issue, evaluating the efficacy of intralesional prednisolone injection after EBD compared to EBD alone in 64 CD patients [25]. The stricture recurrence risk in the combination arm was 4.5 times lower than in the EBD-alone arm at the 12-month follow-up, with clinical remission maintained in 90.7% of patients in the combination arm compared to 65.7% in the EBD_alone arm. In addition, the redilation rate was lower in the combination arm (1.1 ± 0.3 versus 1.44 ± 0.66). Notably, the combination arm observed a better epithelial structure and decreased cellular infiltration and fibrotic deposition at the histological level. No significant difference was reported in terms of perforation and bleeding risk; however, no data concerning stricture location was shown. As a result, the true benefits or harms of intralesional steroid injections after EBD remain unknown, and this technique is not recommended in clinical practice, still representing a missed chance in stricturing CD management.

### 3.3. Endoscopic Balloon Dilation in the Upper Gastrointestinal Tract

Available data on endoscopic management of strictures of upper gastrointestinal tract is still limited, in part due to the lower prevalence of upper gastrointestinal localization and the higher incidence of complex disease in this region, which often requires surgical intervention. Most studies do not provide a separate analysis on the use of EBD for CD-associated strictures of the upper gastrointestinal tract. However, Betterworth et al. analyzed data from multicenter cohort studies involving 94 CD patients who underwent EBD for upper gastrointestinal strictures (107 in the duodenum, 30 in the stomach, 4 in both). Technical and clinical success rates were 100% and 87% respectively. Major complications occurred in 2.9% per procedure, and patients with small-bowel disease location had a higher risk of symptom recurrence and need for redilation. Long-term outcomes were not significantly different fromileocecal stricturesat 24 months (70.5% vs. 75.9% symptom recurrence, 59.6% vs. 73.5% need for redilation, and 30.8% vs. 42.9% surgery) [16]. A recent single-center experience study (2002–2018) investigated the outcomes of 86 patients with benign duodenal stenosis treated with EBD, including 19 patients with CD. This cohort study reported high technical and clinical success rates, which was higher for repeated EBD in CD patients (91.7%)compared to the clinical success rate of repeated EBD for all other aetiologies (74.3%). There were two cases of bleeding (2.3%) and no perforations [26]. Furthermore, patients who underwent aggressive initial dilation were less likely to repeat dilation compared to non-aggressive initial dilation (mean 5.39 versus 4.07 mm more than the estimated caliber of the stricture, *p* 0.07). However, among the 19 patients with CD, 6 still required surgical intervention.

Despite limited evidence available, EBD is gaining traction for duodenal disease [18], demonstrating its potential use in the upper gastrointestinal tract as well. Overall, EBD is considered comparably effective in the short term for both the upper and lower gastrointestinal tract, although its impact on long-term outcomes remains to be evaluated.

### 3.4. Focus on Dilation during Enteroscopy, a Stand-Alone Situation?

CD-related deep small bowel strictures require access through device-assisted enteroscopy, including balloon-assisted enteroscopy (BAE), with outcomes similar to those for strictures reachable by ileocolonoscopy [5,27,28,29]. In a recent meta-analysis of 463 CD patients who underwent EBD for deep small bowel strictures, the overall technical success rate was 94.9%, with a short-term clinical efficacy of 82.3%. During follow-up (median time 25.5 months, IQR 6–53 months), 48.3% of patients reported a recurrence of symptoms, 38.8% were re-dilated, and 27.4% underwent surgery [13]. A nationwide, multicenter, retrospective Japanese study reported surgical conversion rates of 26.0%, 45.6%, and 55.7% at 1, 5, and 10 years post-EBD, respectively [30]; however, this study is based on a large population of patients undergoing dilation with enteroscopy over dilation of the lower gastrointestinal tract (181 over 305), with no differential analysis on deep small bowel strictures. Another prospective, multicenter, Japanese study analyzed EBD through BAE in 95 CD patients, reporting clinical success in 69.5% of the patients, associated with a larger balloon diameter (15.20 ± 1.70 vs. 13.65 ± 2.59 mm, *p* = 0.03) and with a good safety profile (5%, all conservatively managed complications) [27]. Furthermore, a systematic review concluded that dilation of 15 mm or more is a risk factor for perforation [31]; consequently, the generally recommended final target diameter is 12–15 mm in this setting, despite heterogeneous data [32]. Focusing on complications, a systematic review reported incidence rates of severe bleeding and complications requiring surgery between 1.82% and 3.21%, while the incidence rate of perforation ranged from 0–10% in several observational studies [13,27,33]. Possibly due to the increased difficulty and invasiveness of the procedure, higher complication rates have been observed after enteroscopic EBD compared to EBD for ileocecal and gastroduodenal stenosis [15,16].

Overall, due to the lack of specific and comparative evidence on EBD efficacy stratified by stricture localization, these data allow us to affirm that EBD during BAE is comparable to EBD performed in locations achievable by colonoscopy in terms of short-term safety and efficacy. This suggests that EBD has a similar efficacy regardless of stricture location. However, lacking location-specific data, it is still not possible to speculate further, especially on long-term effectiveness and surgery rates.

## 4. Endoscopic Electroincision: Stricturotomy and Strictureplasty

In recent years, there has been increasing interest in stricturotomy (ESt) and strictureplasty (ESTx) as alternatives to EBD in the endoscopic management of stricturing CD. Similar to EBD, these procedures lack standardization in technique and outcome terminology, possibly delaying their exact placement in the IBD landscape. In 2020, the Global Interventional Inflammatory Bowel Disease Group provided detailed guidance and suggested the use of ‘endoscopic electroincision (ES)’ as a unique term for techniques utilizing electrocautery to cut strictured tissue. Electroincision to widen the stenotic lumen (ESt) can be performed using different needle-knives in radial, horizontal, or circumferential orientations, with the possibility of endoscopic clipping after the incision to consolidate the cut with a secondary closure, keeping the lumen wider (ESx) [9]. Endoscopic electroincision is commonly used for papillotomy in endoscopic retrograde cholangiopancreatography, with recent data on esophageal and, more recently, biliopancreatic strictures [34,35,36].

In 2011, Nal et al. published the first case series describing 10 IBD patients with long, fibrotic ileal-pouch strictures refractory to EBD who were treated with ES [37]; the same group from the Cleveland Clinic retrospectively evaluated the efficacy and safety of ES in treating primary and secondary strictures in IBD patients, comparing it with EBD and ileocolic resection [19,20,21,38]. The retrospective study included 50 UC patients with ileal-pouch anastomosis strictures and 35 CD patients (mostly ileocolic anastomosis), with a total of 127 strictures treated with ES, demonstrating a 100% technical success of the procedure [38]. In a median follow-up of 0.9 years, 60.6% of strictures required multiple treatments after the first ES, mostly a subsequent ES (44.9%), an EBD (22.8%), or both combined (11.0%). The cumulative 3-year surgery-free survival rate was 62.0%. Only one patient (0.4% per procedure) experienced perforation, and nine patients (3.3% per procedure) had postprocedural bleeding. The study comparing ES with EBD included CD patients with anastomotic strictures (85.7% ileocolic). It showed a technical success rate of 100% in the 21 patients treated with ES and 89.5% in the 164 patients treated with EBD (*p* = 0.25) [19]. No significant difference in the need for additional endoscopic treatment was found between the two groups (*p* = 0.85). Only two patients (9.5%) in the ES group required subsequent surgery, compared to 55 (33.5%) in the EBD group (*p* = 0.03). It should be noted that the follow-up varied between the two groups, with a median of 0.8 years (IQR: 0.1–1.6) for the ES group and 4.0 years (IQR: 0.8–6.9) for the EBD group (*p* < 0.0001). ES showed a lower risk of perforation than EBD (0% vs. 1.1%), although there were major concerns about bleeding (8.8% vs. 0%). When compared with ileocolic resection (ICR), ES showed comparable surgery-free survival in two different retrospective studies. In the first study on ileocolic anastomosis strictures, 4 out of 35 patients (11.3%) in the ES group and 15 out of 147 patients (10.2%) in the ICR group required subsequent surgery (*p* = 0.83), with a median follow-up duration of 0.8 years for the ES group and 2.2 years for the ICR group (*p* < 0.001) [20]. In the second study on primary distal ileal and ileocecal valve strictures, 2 out of 13 patients (15.4%) in the ES group and 6 out of 32 patients (18.8%) in the ICR group required subsequent surgery (*p* = 0.79) [21]. In this case, the median follow-up duration was comparable: 1.8 years for the ES group and 1.5 years for the ICR group (*p* = 0.84). In both studies, ES showed a lower incidence of major adverse events compared to ICR. Nevertheless, the two groups differed significantly in stricture complexity, with the ICR group having statistically longer and more symptomatic strictures. Even if the majority of the strictures treated with ES in the published studies were located in the ileocolic anastomosis, ES was found to be feasible and safe also for refractory rectal anastomotic strictures [39].

In the IBD setting, low data on ES efficacy is available. Recently, 24 patients with endoscopic non-traversable anorectal/anopouch strictures (18 CD and 4 UC patients) were treated with ES, with a technical success of 100%. However, the mean time to endoscopic reintervention with subsequent ES of 5.3 months. Over a 12.8-month follow-up, two patients (8%) required surgical intervention for refractory stricture disease. No 30-day post-procedure adverse events were reported [40]. ES has been used for deep small bowel strictures in CD (including both the ileum and the jejunum). A multicenter cohort study evaluated the efficacy and safety of BAE-assisted ES for treating these strictures in 28 CD patients with 58 non-passable deep small bowel strictures, resulting in a technical success of 96% and a 1-year cumulative surgery-free rate of 74.8% [41]. Finally, ES is a feasible option for CD-associated anorectal strictures, although evidence is still limited [9].

Overall, ES is a promising procedure for the endoscopic management of stricturing CD, although a slow learning curve could hinder the widespread use of these techniques. Two randomized clinical trials (BEST-CD and DESTRESS) are currently underway to compare EBD and ES in terms of clinical success, need for surgery, and safety with 1-year follow-up in patients with short CD-associated strictures (NCT05521867, NCT05009212).

## 5. The Graveyard of Endoscopic Techniques: Is There Room for a Second Chance?

The history of the endoscopic treatment of stricturing CD has seen the development of several techniques, eventually failing to emerge from the overgrowth into clinical practice for several reasons, including inconsistent clinical trial results, technical difficulties, invasiveness to the patient, poor reproducibility, limited large-scale applicability, and cost. Among the most explored, local injection therapy with anti-tumor necrosis factor (anti-TNF) and endoscopic stents have shown promising results, as well as ongoing novel attempts of improvement to access the IBD treatment landscape.

### 5.1. Anti-TNF Intralesional Injection

Anti-TNF injection has shown promise, although this approach has been nearly abandoned also in the trial setting. In 2008, a pilot study involving 3 CD patients investigated local injections of infliximab (IFX) (90–120 mg) into colonic strictures, resulting in clinical efficacy at 5–8 months in all patients. One patient unresponsive to IFX therapy saw the complete resolution of the stricture after the first local injection and remained symptom-free for 5 months after a second injection. Another patient required additional stricture dilation, while the third one needed five injections every four months [42]. Similarly, another exploratory study from 2014 assessed the efficacy of intralesional injections of 40 mg of infliximab combined with EBD in EBD-refractory small bowel strictures (either primary or anastomotic) in 6 CD patients. Five out of 6 patients underwent serial EBD at 0, 2, and 6 weeks, receiving intralesional infliximab injections after each session. All patients showed a decrease of modified Simple Endoscopic Score for Crohn’s Disease (mSES-CD) by an average of 3 points (reduction in ulcer size, ulcerated surface area, and disease-affected surface area of the distal visible part of the narrowed tract) and improved symptoms, with no adverse effects observed at 6-month follow-up [43]. However, although the authors specify that all enrolled patients had not been previously exposed to infliximab, they do not provide information on any concomitant medical therapies the patients might have been receiving.

A larger, multicenter, randomized, controlled trial on the efficacy of injecting adalimumab into intestinal CD strictures is displayed on trialgov, but it was eventually not published (NCT01986127). While local anti-TNF therapy seems well tolerated, more extended follow-up and larger randomized controlled clinical trials would be necessary to establish its potential benefits. The effectiveness could be significant, moving from a one-time therapy to repeated injection, with anticipated issues concerning quality of life, invasiveness, and health care costs [42,44].

### 5.2. Self-Expanding Metal Stents

Self-expandable metallic stents (SEMS) constitute an effective, non-surgical treatment for neoplastic intestinal obstruction, both as a palliative measure and as a bridge to surgery [45]. SEMS must be fully or partially covered with a plastic film, which prevents colonization by the intestinal mucosa and allows a smooth, delayed extraction. Loras et al. conducted an extensive literature review, describing 19 studies for a total of 65 patients. They identified SEMS as a safe and effective alternative to EBD and surgery for the treatment of short stenosis in CD patients, with possible advantages for complex or longer (>5 cm) strictures [45]. A retrospective study by the same group, involving 17 CD patients treated with SEMS for symptomatic refractory colonic and ileocolic anastomosis strictures, reported a clinical efficacy rate of 64.7%, with patients remaining free of symptoms for an average follow-up period of 67 weeks [46]. Migration occurred in 52% of patients, and there were 4 cases of impaction, with one patient requiring surgery due to proximal stent migration. Another retrospective cohort study involving five patients with anastomotic strictures, where uncovered SEMS were placed for an average of 9.7 months, reported an 80% clinical efficacy rate at a mean follow-up of 28 months [47]. The complication rate was 20% (n = 1), and the four patients who did not require re-intervention showed an average long-term luminal patency of 34.8 months [47]. A prospective cohort study involving 11 patients who received SEMS showed a 60% clinical success rate, with an adverse event rate of 73% (8/11), of which two patients required surgery related to the procedure, and six patients experienced stent migration after an average time of 3 days. As a result, it was concluded that the risk of complications was too high to recommend the routine use of endoscopic metal stents for CD strictures [48]. In a recent study with 21 CD patients, SEMS placement and following removal on day 7 resulted in clinical remission in 88% (14 of 16) of patients during follow-up (3–50 months) [49], with an adverse event rate of 21%, including abdominal pain and asymptomatic stent migration. In a comparative study, 80 CD patients with symptomatic strictures (60% shorter than 4 cm) were randomized to fully-covered SEMS (39) or EBD (41). Despite a similar safety profile, the stent group had a significantly higher proportion of patients requiring new therapeutic intervention at one-year follow-up due to symptoms recurrence (49% vs. 20%) [22]. Notably, the difference in efficacy between EBD and fully-covered SEMS was not significant for strictures over 3 cm (both treatments achieving nearly 65% success) and primary stenosis (respectively 60% and 70%), with a high migration rate representing a potential limiting factor. Given the high rate of stent migration, Branche et al. investigated the Hanarostent stent, a partly covered SEMS with an antimigratory design and an early removal protocol. Following promising results of an exploratory study in 7 CD patients [50], they conducted a larger national study with the same device in 46 CD patients (73.9% with anastomotic stricture, median length of 3.1 ± 1.7 cm). The study observed clinical efficacy in 58.7% of the patients at 26 months follow-up, with no perforations and only three stent migrations reported (6.5%) [51]. Comparative research is ongoing with a randomized clinical trial comparing EBD followed by SEMS placement versus surgical intervention in CD patients with de novo and primary symptomatic stenosis less than 10 cm long [52].

### 5.3. Lumen-Apposing Metal Stents

Lumen-apposing metal stents (LAMS) are short, fully covered metal stents with large flanges at each end to anchor the stent and minimize migration risk [53,54]. Initially designed for draining pancreatic fluid collections, LAMS has been used off-label to manage short-segment luminal strictures [55,56], with several studies showing promising outcomes. Regarding CD patients, evidence is limited: In the light of two case reports on anastomotic strictures showing short-term clinical efficacy and no post-procedural complications [57,58], Hedjoudje et al. evaluated LAMS for lower gastrointestinal anastomotic strictures in 28 patients, including 18 with CD-associated anastomotic strictures. Technical success was achieved in all patients, with clinical efficacy at the last follow-up visit in 85.7% (24/28) of patients. Among the three patients experiencing adverse events, one patient missing his 3-month CT scan experienced failed stent extraction 7 months post-placement and subsequently required surgical resection. Spontaneous asymptomatic stent migration occurred in 47% (13/28) of patients without recurrent symptoms or significant complications [59]. Unfortunately, the authors did not provide a separate analysis for the subgroup of patients with IBD.

### 5.4. Biodegradable Stents

Considering the high migration rates and the need for follow-up endoscopy for the removal of SEMS, biodegradable stents (BDS) have been developed and primarily evaluated for esophageal strictures, yielding promising outcomes [60,61,62]. However, they are not approved for bowel strictures. BDS exert a constant radial force for approximately 4–5 weeks to treat the underlying esophageal disease, while progressive hydrolysis-mediated self-degradation prevents tissue overgrowth and leads to dissolving within 12 weeks [63]. A prospective study evaluated polydioxanone monofilament stents, which provide 6–8 weeks of radial force before degradation, in a cohort of 11 patients with benign small and large intestinal stenosis naïve to EBD [64]. The study showed a technical success rate of 91%, with no adverse events other than early stent migration in three patients. However, few studies, mainly case reports and series, have investigated BDS in stricturing CD. Rejchrt et al. reported successful BDS insertion for small and large bowel stenosis in 10 out of 11 CD patients, with early stent migration (2 days to 8 weeks) observed in three patients [64]. Karstensen et al. documented the case of a 52-year-old man with CD, successfully treated with a custom-made biodegradable polydioxanone monofilament stent (15 cm) for a 12 cm small bowel stricture, remaining symptom-free at 3-month follow-up [65]. However, a subsequent study by the same group involving six CD patients with intestinal stenosis at various locations (duodenal bulb, ileocolic anastomosis, afferent limb of a J-pouch, and sigmoid colon) refractory to EBD and treated with biodegradable stents, reported clinical success in only one patient. Failures were attributed to mucosal overgrowth in two patients, stent migration in one patient, and stent collapse in another [66].

## 6. New Techniques and Future Scenarios for Stenosis and Fibrosis Treatment in IBD Involving Pathogenesis and Molecular Pathways

Future scenarios for stenosis treatment in IBD are likely to involve a combination of minimally invasive procedures and targeted therapies. As we improve our knowledge of fibrosis pathogenesis and stenosis development in IBD, novel therapeutic opportunities are emerging, combining the identification of new molecular targets with the development of innovative local delivery systems.

The advent of single-cell transcriptomics has better defined the transcriptional profile of fibroblasts in CD and ulcerative colitis (UC) [67,68]. Targeting fibroblasts at a cellular and molecular level has been a promising subject of investigation. For instance, when adding pirfenidone, an agent approved for treating idiopathic pulmonary fibrosis, to fibroblasts isolated from patients with stricturing CD, their function and proliferation are inhibited via downregulation of the transforming growth factor beta 1 (TGFβ1) pathway [69]. Parallelly, several small experimental molecules have been tested as potential inhibitors of the main molecular pattern of fibrosis, with still inconclusive and inconsistent results [70,71]. Moreover, stem cell manipulation and administration offer a promising scenario with a constantly increasing body of evidence. Human adipose-derived mesenchymal stem cells, pretreated in vivo with interferon (IFN)γ and kynurenic acid, ameliorate intestinal injury in a fibrosis rat model [72]. Both prophylactic and therapeutic treatment with bone marrow-derived mesenchymal stem cells improve fibrosis and reduce collagen deposition, via interleukin(IL)-1beta, IL-6, and IL-13 downregulation, and IL-10 upregulation [73]. In experimental colitis mice, mesenchymal stem cells can reduce the thickness of submucosa/muscularis propria, as well as collagen deposition. Furthermore, in human primary intestinal myofibroblasts mesenchymal stem cells reduce the TGF-β1-induced fibrogenic activation [74]. Antibodies targeting proteins involved in collagen remodeling have been identified as a possible therapeutic strategy as well; the inhibition of matrix metalloproteinase-9 (MMP-9), a type IV collagenase overexpressed in fistulizing and stricturing CD, led to reduced collagen deposition in heterotopic xenograft models of intestinal fibrosis [75]. Among other unexplored therapeutic targets is teduglutide, already approved for the treatment of short bowel syndrome. Teduglutide has shown a reduction in fibrogenesis and improved fibrinolysis from the first week after surgery in a murine model of ileal resection and anastomosis [76,77]. More recently, the anti-fibrogenic effects of glucagon-like peptide 2 (GLP2) have also been demonstrated in the liver of a murine model of cholangitis [78].

Novel biomarkers and pharmacological agents will play a crucial role in enhancing treatment outcomes, possibly involving novel local delivery systems to allow tissue-targeted therapy and minimize side effects. Over the past two decades, there was a slow but consistent understory of research and development of a large number of nanoparticles (NPs) in IBDs. NPs are polymeric fine units with at least one dimension up to 100 nm, which can be built to reach an intended target, be detected with a molecular imaging technique and further engineered to locally deploy a certain agent. They employ several mechanisms of activation, including charge-mediated targeting, micro-environment–triggered release targeting, and ligand-mediated targeting [79,80]. Nanomedicine combined with endoscopy has built up a solid background in ex vivo and in vivo preclinical settings, with minimal preliminary clinical data concerning esophageal and biliary cancers [79,81,82]. However, there has been no significant impact on clinical practice, partly due to safety concerns, heterogeneity of NPs, practical application issues, and variability in study designs. The dual potential of NPs as drug-eluting and detectable nano vehicles represents an exciting perspective in IBD, potentially leading to dose-controlled and selective insite bioavailability of a drug or a therapeutic intervention targeting inflamed mucosa [83], eventually within strictures. Among intestinal delivery systems, another remarkable product recently developed is a biocompatible hydrogel amenable to endoscopic application (CoverGel), showing intriguing preclinical data. In experimental colitis mice, when comparing CoverGel + IFX versus subcutaneous IFX alone, similar efficacy on inflammation was observed, with significantly lower levels of antibodies to infliximab in the CoverGel group [84].

## 7. Conclusions

In the last decades, endoscopy has been engaging stricturing CD as a minimally invasive approach aiming to delay and prevent surgery and promote a superior quality of life. Although EBD is indeed the standard of care for short strictures, the extensive data available is burdened by wide variability in dilation technique and study settings, leaving several unsolved issues mostly concerning long-term outcomes. In this scenario, novel endoscopic techniques are emerging, with still preliminary conflicting results and active research ongoing. Moreover, the new insight into the molecular patterns of fibrosis paves the way for future developments, possibly aided by new endoscopic local drug-eluting systems.

More long-term, head-to-head prospective studies are essential to eventually move endoscopic management out of the gray area between a bridge to surgery and a bowel-sparing technique.

## Figures and Tables

**Table 1:** Comparative studies currently available on the management of CD-associated strictures.

	Study Design	Strictures Location	Treatment and No of Pts	Technical Success Rate	Long-Term Outcomes (Years fu)	Adverse Events, %
[17] 2017	Retrospective	Ileocolic anastomosis	176 EBD 131 surgery	-	average time to surgery/re-surgery delayed by 6.45 years in EBD group	1.1 (perforation) 8.8 (~infection)
[18] 2024	Retrospective	Duodenal	30 EBD 18 surgery	-	2.96 years recurrence-free 6.31 years recurrence-free, *p* = 0.01	0.74 16.67
[19] 2018	Retrospective	Anastomosis (85.7% ileocolic)	21 ES 164 EBD	100% 89.5%	9.5% surgery (0.8) 33.5% re-surgery (4)	8.8 (bleeding) 1.1 (perforation)
[20] 2019	Retrospective	Ileocolic anastomosis	35 ES 147 ICR	-	11.3% surgery (0.8) 10.2% re-surgery (2.2), *p* = 0.83	10.2 (~bleeding) 31.9 (~ileus)
[21] 2020	Retrospective	Distal ileum, ileocecal valve	13 ES 32 ICR	100% 100%	15.4% surgery (1.8) 18.8% re-surgery (1.5), *p* = 0.79	6,9 (perforation) 25 (~infection)
[22] 2022	Randomized trial	-	41 EBD 39 FCSEMS	-	80% no re-intervention (1) 51% no re-intervention (1), *p* = 0.0061	2 (perforation) 3 (perforation)

ES, endoscopic electroincision; EBD, endoscopic balloon dilation; FCSEMS, fully-covered self-expandable metallic stents; fu, follow-up ICR, ileocolic resection; *p*, *p*-value; pts, patients; ~, most frequent.

## Data Availability

Not applicable.
